# Ectopic pancreatic-type malignancy presenting in a Meckel's diverticulum: a case report and review of the literature

**DOI:** 10.1186/1477-7819-7-54

**Published:** 2009-06-22

**Authors:** Hoey C Koh, Blaithin Page, Catherine Black, Ian Brown, Stuart Ballantyne, David J Galloway

**Affiliations:** 1 Department of Clinical Surgery, Gartnavel General Hospital, 1053 Great Western Road, Glasgow G12 0YN, UK; 2 Department of Pathology, Crosshouse Hospital, Kilmarnock KA2 0BE, UK; 3 University of Glasgow, Department of Pathology, Western Infirmary, Dumbarton Road, Glasgow G11 6NT, UK; 4 Department of Radiology, Gartnavel General Hospital, 1053 Great Western Road, Glasgow G12 0YN, UK

## Abstract

**Background:**

Neoplasms arising from Meckel's diverticulae reported in the literature are mainly carcinoid tumours, gastrointestinal stromal tumours, and gastric or intestinal adenocarcinomas.

**Case presentation:**

We describe a 50-year-old man who presented with rectal bleeding and anaemia, later found to be caused by a pancreatic adenocarcinoma arising from ectopic pancreatic tissue in a Meckel's diverticulum. The tumour was unfortunately highly aggressive, and the patient passed away within 5 months of symptom onset.

**Conclusion:**

We believe this is the first case of pancreatic adenocarcinoma in a Meckel's diverticulum to be reported in the literature. The diagnosis of Meckel's should be considered in patients with acute gastrointestinal complaints; when found incidentally at laparotomy, it should be carefully examined for any gross abnormality and resection should be considered.

## Background

Meckel's diverticulum is the most common congenital anomaly of the gastrointestinal tract, affecting approximately 2% of the population [1,2]. It is a true diverticulum occurring on the anti-mesenteric border of the distal ileum, typically within 100 cm of the ileo-caecal valve. Neoplasms arising in Meckel's diverticulae are uncommon. Those reported in literature are mostly carcinoid tumours, followed by gastrointestinal stromal tumours, leiomyosarcomas and gastric or intestinal adenocarcinomas. There has been one case of intraductal papillary mucinous adenoma arising from ectopic pancreatic tissue in Meckel's diverticulum [3]. To our knowledge, pancreatic adenocarcinoma arising in a Meckel's diverticulum has never been reported in the literature. In our case report, we describe a patient who was found to have such tumour. The clinical and pathological aspects of this case are reviewed as well as the related literature.

## Case presentation

A 50-year-old man presented with a 4-week history of rectal bleeding with associated dyspnoea on exertion. His past medical history was unremarkable and there was no significant family history. He was a non-smoker and a social drinker. Physical examination including a digital rectal examination was unremarkable.

Initial investigations revealed a hypochromic, microcytic anaemia with a haemoglobin level of 8 g/dl and a ferritin level of 4 μg/L. Biochemical assessments of liver and renal functions were normal. Gastroduodenoscopy was normal and colonoscopy only revealed an incidental 2 mm benign tubular adenoma in the rectum.

**Figure 1:**
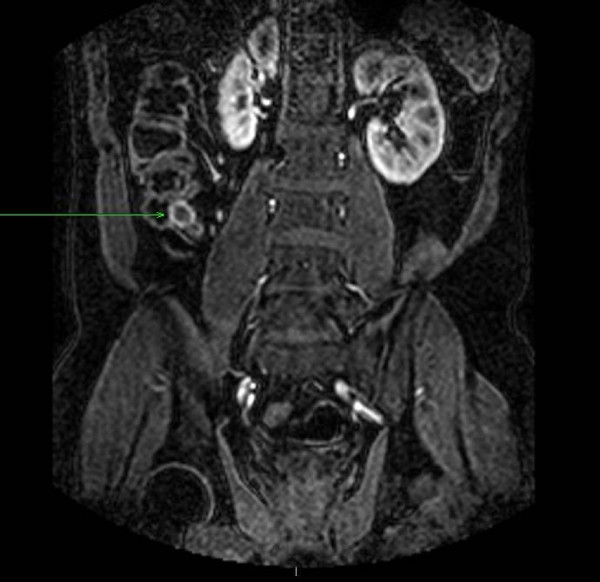
**MRI enteroclysis**. Green arrow shows a 22 mm peripherally enhancing soft tissue lesion in the right iliac fossa.

**Figure 2:**
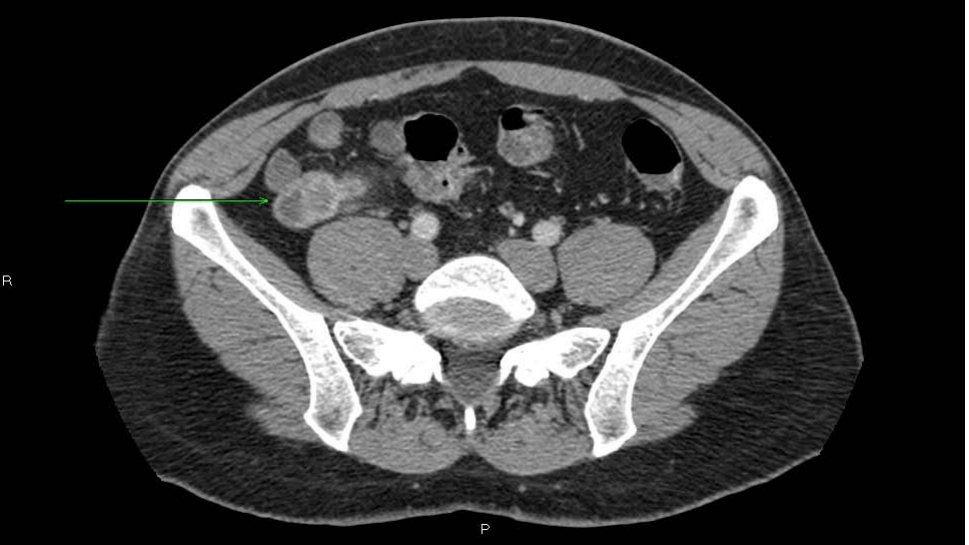
**CT enterography**. Green arrow shows a mass lesion arising from either the appendix or a Meckel's diverticulum.

Cross-sectional imaging was carried out. A MRI enteroclysis (Figure 1) revealed a 22 mm peripherally enhancing soft tissue lesion in the right iliac fossa, and a CT enterography (Figure 2) suggested that the mass lesion was arising either from the appendix or a Meckel's diverticulum.

A laparotomy was carried out via a grid iron incision. A Meckel's diverticulum was found to be adherent to the tip of the appendix in the right iliac fossa. There was a primary tumour arising from the Meckel's diverticulum and multiple sub-centimetre peritoneal deposits in the adjacent visceral peritoneum. These deposits were further distributed in the parietal peritoneum on the right side of the true pelvis and as tiny granular deposits in the greater omentum. A Meckel's diverticulectomy and *en bloc *appendicectomy was carried out and an omental deposit was sampled for histological assessment.

Gross examination of the specimen revealed a 40 mm diameter tumour arising from the mucosal aspect of the bowel wall of the Meckel's diverticulum. The tumour directly involved the peritoneal wall, and had invaded the serosal surface of the small bowel. The resection margins and the adhered appendix were tumour-free. A single omental deposit submitted separately was also involved by the tumour.

**Figure 3:**
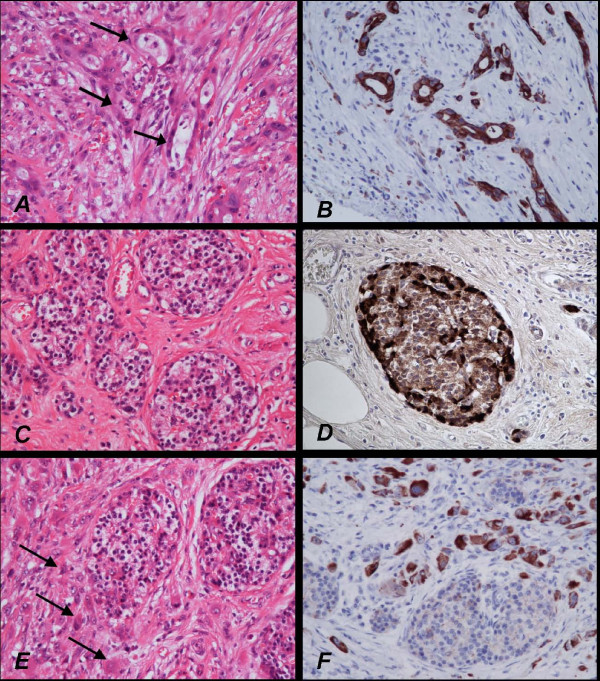
**Histology and immunoprofile of pancreatic type adenocarcinoma**. The malignant glands *(arrows in A) *are highlighted by cytokeratin expression *(B)*. The poorly differentiated single tumour cells infiltrating the tissue surrounding the ectopic pancreatic islets are difficult to identify with the conventional haematoxylin and eosin (H&E) section *(arrows in C)*, but are highlighted by cytokeratin *(D)*. The islets *(E) *express neuroendocrine markers, and specific pancreatic islet cell markers insulin *(F) *and glucagon.

Histology of the tumour (Figure 3) showed that it was composed of scattered small glandular structures and also scattered bizarre single tumour cells. The tumour cells expressed cytokeratin CK7, and CA 19.9. There was no expression of CK20, CDX2 or CEA. These findings support an upper gastrointestinal/pancreatico-biliary origin. Associated with the tumour were nests of cells with a pancreatic islet morphology, which expressed general neuroendocrine markers (CD56, synatophysin and chromogranin), and specific islet cell markers insulin and glucagon. Carcinoid markers serotonin and gastrin were negative. These appearances are consistent with the tumour being a pancreatic-type adenocarcinoma arising from ectopic pancreatic tissue in a Meckel's diverticulum.

The patient made good post-operative recovery. He was discharged following multi-disciplinary discussion among surgeons, radiologists and oncologists, with detailed out-patient follow-up arrangements in place, including an outpatient appointment with the pancreatic oncology specialist within a week of discharge.

**Figure 4:**
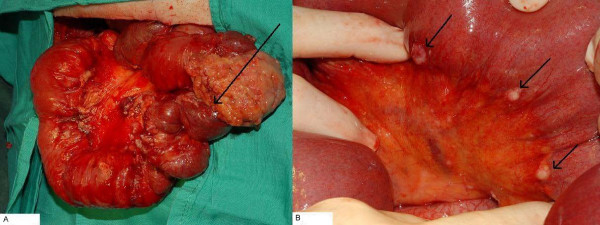
**4a shows a dramatic increase in the volume of tumour encountered in the 2^nd ^operation, merely 2 weeks after the 1^st ^operation of tumour resection**. **4b **shows a significant increase in the size, number and extent of peritoneal deposits.

Two weeks following discharge however, he developed food intolerance and small bowel obstruction was confirmed on CT scan. A second laparotomy was carried out and a dramatic increase in the volume of tumour was encountered (Figure 4a) with a significant increase in the size, number and extent of peritoneal deposits (Figure 4b). There was no single point of obstruction in the distal small bowel and in view of the encasement of distal ileum and proximal colon together with local mesenteric infiltration, an ileo-transverse colonic bypass was performed to relieve the obstruction.

Subsequent management was palliative and the rapid tumour progression continued until his death some 6 weeks after the initial operation.

## Discussion

Meckel's diverticulum was first described by Fabricus Hildanus in 1598 [2], and was later named after the German anatomist, Johann Friedrich Meckel, who described its embryological origin in 1809 [2]. It is the vestigial remnant of the vitello-intestinal duct, which acts as a communicating tract between the embryonic yolk sac and its primitive mid-gut in the first few weeks of development. Failure of complete obliteration of the vitello-intestinal tract results in a variety of congenital defects, of which Meckel's diverticulum is the commonest anomaly [1].

Patients with Meckel's are usually asymptomatic, and the diverticulae are invariably discovered incidentally at autopsy, laparotomy or laparoscopy. These patients have a 2–4% lifetime risk of developing complications from it [1,2,4]. Complications from Meckel's usually arise from its underlying mucosa, 50% of which are ectopic mucosae such as gastric mucosa (17.9% – 40%), pancreatic tissue (5–16%), and less commonly, duodenal, colonic and biliary tissue [1,2]. The complications are commonly intestinal obstruction, intussusception, inflammation, haemorrhage and less commonly, perforation, herniation and neoplasm [1,2].

Heterotopic pancreatic tissue itself is uncommon, with reported frequency between 0.55 to 13.7% [5]. It is the presence of pancreatic tissue which lacks anatomical and vascular continuity with the pancreas. It is usually found in the stomach, duodenum and upper part of jejunum, less commonly in the Meckel's, ileum, biliary system, and even spleen. Similar to Meckel's diverticulum, ectopic pancreatic tissues are usually asymptomatic and are found incidentally; they too can occasionally cause symptoms such as bleeding, inflammation, abdominal pain and rarely malignant changes. Not unexpectedly, complications are usually found in the stomach and duodenum. To our knowledge, there is only a case reported in the literature of a benign intraductal papillary mucinous adenoma arising from ectopic pancreatic tissue in a Meckel's diverticulum [3], and ours is the first malignant ectopic pancreatic adenocarcinoma in a Meckel's diverticulum to be reported in the literature. Neoplasms arising from Meckel's are quoted to be 3.2% [6]; the majority of Meckel's tumours are carcinoid tumours (33%), followed by gastrointestinal stromal tumours (GIST), benign leiomyomas and less commonly gastric or intestinal adenocarcinomas. Tumours in Meckel's present non-specifically with gastrointestinal complaints such as bleeding, obstruction, inflammation or perforation. The suspicion of a Meckel's is however often not thought of at the initial stage of patient management, and the diagnosis of Meckel's is quite challenging and it is not infrequently overlooked on radiological imaging unless one is actively looking for it, the tumours tend not to be diagnosed till late and sometimes, as in our case, at such an advanced stage that the delay in intervention proves to be futile.

The authors however, are not advocating incidental diverticulectomy in every patient found to have a Meckel's. Soterro and Bill have reported that up to 800 incidental diverticulectomies are required in order to save one life [4], and the procedure itself has complication rates of up to 8%, including a mortality rate of 1.2%. This outweighs the 2–4% lifetime risk of developing complications from Meckel's. Dumper et al [6] therefore recommend a case-by-case approach with factors favouring resection like younger age at presentation, palpable or visual abnormality of the Meckel's, previous symptoms which might be caused by the Meckel's such as obstruction or bleeding. A case report by Carpenter et al [7], who reported on carcinoid tumours in Meckel's, has stated that as such tumours in Meckel's are rare with unpredictable natural history, it is difficult to determine on any standard treatment. They have suggested performing en bloc resection for small tumours, and debulking resection as well as palliative radiotherapy and/or systemic chemotherapy for widespread unresectable disease.

## Conclusion

Although Meckel's and its complications are not common, the possibility of a Meckel's diverticulum and its potential complications should be considered when faced with a common gastrointestinal complaint and negative initial investigations. When found incidentally at laparotomy or laparoscopy, it should be carefully examined for any gross macroscopic abnormality and resection should be considered, especially in young male patients who are more likely to develop complications from it or patients who might have had previous symptoms attributable to it.

## Consent

Written informed consent was obtained from the patient for publication of this case report and accompanying images. A copy of the written consent is available for review by the Editor-in-Chief of this journal.

## Competing interests

The authors declare that they have no competing interests.

## Authors' contributions

HK and BP reviewed the literature and wrote the case presentation. CB and IB described the histological findings and confirmed and edited the manuscript. SB provided radiological images and confirmed the manuscript. DG conceived the case report, helped draft and revised the manuscript. All authors read and approved the final manuscript.
